# Synergistic chemotherapy and immunomodulatory effects of Quercetin in cancer: a review

**DOI:** 10.3389/fimmu.2025.1547992

**Published:** 2025-05-26

**Authors:** Hongyang Deng, Fengxian Wei, Wei Han, Yongfang Li, Xiaodong Xu, Lingyi Zhang, Youcheng Zhang

**Affiliations:** ^1^ Department of General Surgery, Hepatic-biliary-pancreatic Institute, The Second Hospital & Clinical Medical School, Lanzhou University, Lanzhou, Gansu, China; ^2^ Department of Liver Disease, The Second Hospital & Clinical Medical School, Lanzhou University, Lanzhou, Gansu, China

**Keywords:** quercetin, cancer, 5-fluorouracil, doxorubicin, nephrotoxicity, neurotoxicity, immune cells

## Abstract

Cancer is a significant public health problem worldwide, and its morbidity and mortality are challenging to improve, which is an important obstacle to prolonging life expectancy. Cytotoxic drugs have been used in anti-cancer therapy since the 1940s. They play an important role in tumor therapy. However, drug resistance and systemic toxicity often limit its application. Combination or synergistic chemotherapy can promote therapeutic effects and reduce toxicity. Quercetin (QUE) is a natural flavonoid widely found in fruits and vegetables. It has anti-cancer, anti-inflammatory, antioxidant, and neuroprotective properties. An increasing number of studies have found that the combination of QUE and chemotherapy drugs has a chemosensitization effect. To a certain extent, it can inhibit the side effects of chemotherapeutic drugs, such as nephrotoxicity, cardiotoxicity, reproductive toxicity, and neurotoxicity, which has attracted great attention. The immune system plays a significant role in tumor development. Notably, several studies have revealed that QUE plays an immunomodulatory role by promoting the differentiation of anti-cancer immune cells and inhibiting immune checkpoint expression. In conclusion, current studies have emphasized the potential of QUE in chemosensitization, reduction of toxic side effects, and enhancement of the anti-cancer immune response. However, more preclinical and clinical cohort studies are needed to determine QUE’s efficacy, mechanism, optimal formulation, and long-term effects in synergistic chemotherapy and immunomodulatory effects.

## 1 Introduction

Cancer is a complex genetic disease, and multiple genetic and environmental factors have influenced its occurrence and development. It arises from dysregulation of cell proliferation and concomitant genomic modifications (1). Unfortunately, cancer prevalence continues to increase to the extent that it has become the second leading cause of death worldwide after heart disease (2). About 20 million new cancer cases were reported in 2022, including nearly 9.7 million deaths (3). With the development of cancer research, our understanding of the biological characteristics and treatment options for cancer continues to evolve. Several therapies have been used to treat cancer, including surgery, immunotherapy, radiotherapy, chemotherapy, endocrine therapy, and various RNA molecules (4). Among these, chemotherapy is a relatively effective option, particularly for advanced cancers. Unfortunately, chemotherapy cannot wholly eradicate all cancer cells, and cancer cells may reappear within a short period (5). Chemotherapeutic drugs also have a significant impact on normal cells, causing inevitable damage to the body during chemotherapy, such as alopecia, gastrointestinal toxicity, nephrotoxicity, and neurotoxicity (6). Therefore, improving the sensitivity to chemotherapy drugs and reducing side effects is a continuing concern in cancer drug therapy.

**Figure 1:**
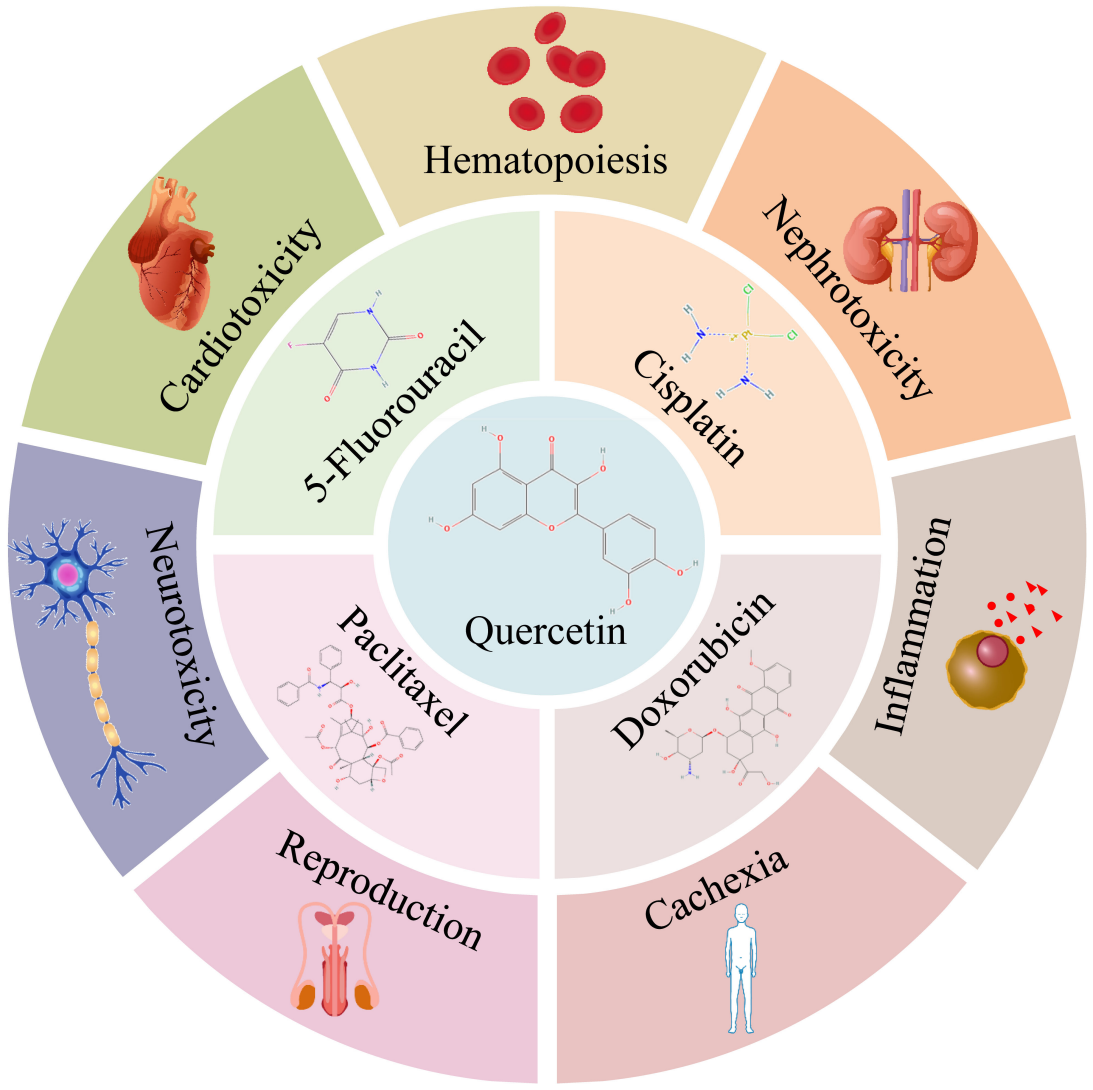
QUE synergizes with 5-Fluorouracil, cisplatin, doxorubicin, and paclitaxel and alleviates side effects of chemotherapy. [Chemical structures were obtained from Pubchem (20)].

Quercetin (QUE) is a flavonol compound that is insoluble in water but soluble in alcohol and lipids (7). It is rich in grapes, cherries, buckwheat, berries, Onions, citrus fruits, and so on (8). Many *in vitro* and *in vivo* studies have demonstrated the effects of QUE in cancer treatment. It has been reported (9) that QUE has many anti-cancer properties, including the ability to inhibit proliferation, anti-angiogenesis, induce apoptosis, and inhibit mitotic processes (10), as well as many benefits such as antioxidant (7, 11), anti-inflammatory (12), neuroprotection (13), antimicrobial (14), anti-allergy (15), and immune regulation functions. QUE shows strong potential for adjuvant chemotherapy because of its low toxicity and multi-target nature (16). Many studies have shown the potential of QUE to prevent drug resistance and sensitize cancer cells to chemotherapeutic agents by modulating key factors associated with tumor growth and progression, such as inhibition of anti-apoptotic proteins, activation of pro-apoptotic proteins, and inhibition of cell cycle proteins (16). In addition, a growing body of evidence highlights the potential of QUE as a natural compound to prevent chemotherapy-induced cardiotoxicity (17), nephrotoxicity (18), and neurotoxicity (19). **Figure 1** illustrates the chemotherapeutic drugs that synergize with QUE and the side effects mitigated by QUE.

Tumor development and progression depend on the interactions between cancer cells and multiple cell types surrounding the tumor, constituting the tumor microenvironment (TME) (21). The immune system is an important factor that controls the occurrence and development of tumors (22) and is an emerging field of tumor therapy. Cancer cells usually must evade immune surveillance to grow and metastasize indefinitely (22). Therefore, developing immunogenic therapies to reverse immune surveillance and activate anti-cancer responses is promising. Several compounds have been shown to interact with subsets of immune cells to stimulate anti-cancer immune responses (23). It has been reported (24) that QUE promotes activation of the host immune system and anti-cancer immune response, inhibiting tumor growth and possibly a new anti-cancer immunotherapy.

This review summarizes the data obtained from various studies to explore the benefits of QUE in combination with various chemotherapeutic agents, which manifest in two aspects: enhanced chemotherapy sensitivity and reduced toxic side effects of chemotherapy. The effect of QUE on tumor immunity is also discussed. This review seeks to contribute to the advancement of QUE and improved treatment outcomes for cancer patients.

## 2 The chemosensitization effect of QUE in cancer chemotherapeutic

The mechanisms of chemotherapeutic drugs are complex and include effects on DNA structure, inhibition of nucleic acid synthesis, DNA replication, and interference with mitotic tubulin (6). The mechanisms of acquired drug resistance in tumor cells usually manifest in several aspects (25): (1) The balance between drug absorption and excretion is disrupted, decreasing intracellular drug levels. (2) As a protective process, autophagy inhibits tumor cell apoptosis. (3) Induction of immunosuppression. Combining chemotherapy drugs with QUE has shown synergistic or sensitizing effects and higher cytotoxic effects on tumor cells.

### 2.1 5-Fluorouracil

5-FU is a metabolic inhibitor that interferes with nucleic acid metabolism by inhibiting cancer cells’ thymidylate synthase (TS) activity, blocking their DNA synthesis, and inhibiting their proliferation. Since 1957, it has been widely used for various types of cancer, such as colorectal cancer (CRC), breast cancer (BC), and gastric cancer (GC) (26). Despite its many advantages, its clinical application is limited because of the progressive development of drug resistance after chemotherapy (26). Therefore, new therapeutic strategies to suppress drug resistance and improve the response to 5-FU are urgently needed.

The 5-FU-resistant cells express high levels of TS. 5-FU administration for 24 h can increase the expression level of bound TS in HCT116 and HT29 CRC cells and inhibit the expression of p53, thereby tolerating 5-FU (27). The combination of QUE and 5-FU significantly attenuated 5-FU-induced upregulation of TS expression and enhanced the 5-FU effect by inducing mitochondrial dysfunction and increasing the expression levels of p53 (28). p53 is closely related to 5-FU resistance, and its mutation reduces apoptosis and cell cycle inhibition (29). Another study found that QUE could activate p53 expression in CO115 and HCT15 CRC cells, rendering these two cells sensitive to 5-FU (30). Das et al. used QUE and 5-FU to construct chitosan nanoparticles, which induced apoptosis and cell cycle arrest at the G0/G1 phase in HCT116 CRC cells by activating the p53/p21 axis (31). In addition, Li et al. found that QUE enhanced the sensitivity of HCT116 and SW480 cells to 5-FU by inducing autophagy and up-regulating the expression of Drp-1, resulting in mitochondrial fragmentation (32). Tang et al. constructed a 5-FU-resistant HCT116 cell line and found that QUE reversed 5-FU resistance by inhibiting the Nrf 2/HO-1 pathway (33). In addition, QUE combined with 5-FU enhanced the inhibitory effect of 5-FU on the proliferation of HCT116 and Caco-2 cells by down-regulating miR-27a/Wnt/β-catenin signaling and up-regulating SFRP1 expression (34). NLRP3 is an inflammasome, and its activation is closely related to tumorigenesis and tumor-related inflammation (35). Lee et al. found that after resistin-induced resistance to 5-FU in CRC cells, the phosphorylation level of p-ERK increased to promote NLRP3 expression, indicating that NLRP3 expression increased the resistance of HCT116 cells to 5-FU (36). Therefore, QUE could enhance the cytotoxic effect of 5-FU on HCT116 cells by inhibiting ERK/NLRP3 (36). QUE synergistically enhanced the sensitivity of HT-29 CRC cells to 5-FU by up-regulating the expression of p38, PTEN, Bax, and p53, but down-regulating the expression of AKT, mTOR and bcl-2 (37). As a prodrug form of 5-FU, capecitabine is used as monotherapy in a variety of advanced or metastatic cancers. When combined with capecitabine, QUE increased the killing effect of capecitabine on COLO320 CRC cells and MCF-7 BC cells, and arrested the cell cycle by inducing mitochondrial depolarization and promoting the expression of Caspase-3, while it had no cytotoxicity on normal mouse fibroblast 3T3-L1 cells (38).

In BC, 5-FU combined with QUE further promoted the apoptosis of MCF-7 cells by increasing the expression of Bax, Caspase-9, and p53 genes and decreasing the expression of bcl-2 genes, indicating that QUE could enhance the sensitivity of 5-FU to BC (39). In addition, the combination of QUE and 5-FU had a better inhibitory effect on the migration ability and the expression of MMP2 and MMP9 in highly invasive MDA-MB-231 cells than 5-FU alone (40). In addition, QUE enhanced the cytotoxic effect of 5-FU on EC9706 and Eca109 esophageal cancer cells by inhibiting NF-κB (41). Roman initially found (42) that QUE could enhance the cytotoxic effect of 5-FU on A375 melanoma cells but did not explore the mechanism. To explore the relationship between QUE and 5-FU pharmacokinetics, Tavakoli et al. intraperitoneally injected QUE and 5-FU into rats. Subsequently, blood samples were collected to evaluate the pharmacokinetic parameters using high-performance liquid chromatography, and it was found that QUE significantly increased the plasma concentration of 5-FU and improved the availability of 5-FU (43).

In conclusion, the present study suggests that QUE sensitizes CRC and BC cells to 5-FU by regulating the expression of apoptotic proteins (**Table 1**). It has been established that QUE positively affects the combination of 5-FU, but the specific mechanism deserves further exploration. We found that the sensitivity to 5-FU is related to the inflammasome NLRP3, which is an interesting topic. In addition, current studies have focused on the role of QUE combined with 5-FU in CRC and BC, but 5-FU is suitable for a variety of tumors such as GC (44) and hepatocellular carcinoma (HCC) (45). Subsequent studies are needed to explore the role of QUE combined with 5-FU in these tumors.

**Table 1:** Mechanism of QUE enhancing the sensitivity of different tumor cell lines to 5-FU.

Cancer type	Cell line	Mechanism	Reference
Colorectal cancer	HCT116, HT29	↓TS ↑p53	(28)
Colorectal cancer	CO115, HCT15	↑p53	(30)
Colorectal cancer	HCT116	↑p53, p21	(31)
Colorectal cancer	HCT116, SW480	↑Drp-1	(32)
Colorectal cancer	HCT116	↓Nrf 2/HO-1	(33)
Colorectal cancer	HCT116, Caco-2	↓miR-27a/Wnt/β-catenin ↑SFRP1	(34)
Colorectal cancer	HCT116	↓p-ERK, NLRP3	(36)
Colorectal cancer	HT-29	↑p38, PTEN, Bax, p53 ↓AKT, mTOR, bcl-2	(37)
Colorectal cancer, Breast cancer	COLO320, MCF-7	↑Caspase-3	(38)
Breast cancer	MCF-7	↑Bax, p53, Caspase-9 ↓Bcl-2	(39)
Breast cancer	MDA-MB-231	↓MMP2, MMP9	(40)
Esophageal cancer	EC 9706, Eca 109	↓NF-κB	(41)

“↑” represents QUE’s promoting effect. “↓” represents QUE’s inhibiting effect.

TS, thymidylate synthase.

### 2.2 Doxorubicin

Doxorubicin (DOX) is one of the most effective chemotherapeutic drugs for BC treatment. It is a naturally occurring anthracycline antibiotic that prevents DNA replication by inhibiting DNA topoisomerase II (46). Drug resistance is also one of the main reasons for DOX treatment failure, leading to poor prognosis and survival (47). Solving the drug resistance of DOX is a new hope for cancer treatment.

Li et al. found that non-toxic doses of QUE enhanced the toxic effect of DOX on MCF-7 BC cells by up-regulating PTEN and inhibiting p-AKT phosphorylation (48). QUE also reversed DOX resistance in the MCF-7/DOX-resistant BC cell line by down-regulating the SNAI2, PLAU, and CSF1 genes (49). Triple-negative breast cancer (TNBC) has a poor response to endocrine and anti-HER2 therapies, and chemotherapy is the primary treatment in addition to surgical resection. A study reported that QUE could improve DOX and cyclophosphamide (CTX) chemosensitivity by inhibiting ROS production and the p-ERK1/2 pathway in TNBC cells MDA-MB-231 (50). In HOS and MG-63 osteosarcoma cells, the combination of QUE and DOX was shown to reverse DOX resistance in osteosarcoma by increasing mitochondrial biogenesis (51). Similarly, QUE enhanced the killing effect of DOX on P388 leukemia cells by inhibiting the expression of NF-κB, p65, and Bcl-2 but by activating Bax and Caspase-3 (52). For the acquired DOX-resistant PC3 prostate cancer cell line, QUE was found to reverse its resistance by inhibiting c-Met and PI3K/AKT signaling pathways (53). In SMMC7721 and QGY7701 HCC cells, QUE promoted the expression of p53, inhibited Bcl-xl, and up-regulated the expression of Caspase-3/9, which enhanced the effect of DOX on promoting apoptosis (54). Li et al. constructed a hyaluronic acid (HA)-modified zeolitic imidazolate framework for loading DOX and QUE, prioritizing targeting HEPPG2/DOX resistant cells overexpressed CD44. Moreover, the released QUE reshaped the TME by reducing the content of collagen and α-SMA in tumor tissues and significantly increased the anti-cancer effect of DOX (55). In addition, QUE loading with lectin-stabilized polymeric micelles enhanced DOX growth inhibition in CT26 CRC cells and reduced cardiotoxicity (56).

Many studies have reported an augmentation effect of QUE on DOX chemotherapy regimens (**Table 2**). The known mechanism of QUE increases the apoptotic process of tumor cells, but this mechanism is complex, and further studies are needed to confirm this.

**Table 2:** Mechanism of QUE enhancing the sensitivity of different tumor cell lines to DOX.

Cancer type	Cell line	Mechanism	Reference
Breast cancer	MCF-7	↑PTEN ↓P-AKT	(48)
Breast cancer	MCF-7	↓SNAI2, PLAU, CSF1	(49)
Breast cancer	MDA-MB-231	↓p-ERK1/2	(50)
Leukemia	P388	↓NF-κB, p65, Bcl-2 ↑Caspase-3, Bax	(52)
Prostate cancer	PC3	↓c-met, PI3K/AKT	(53)
Hepatocellular carcinoma	SMMC7721, QGY7701	↑p53, Caspase-3/9 ↓Bcl-xl,	(54)

“↑” represents QUE’s promoting effect. “↓” represents QUE’s inhibiting effect.

### 2.3 Cisplatin

In 1965, Cisplatin (CIS) was found to inhibit cell division. It blocks the synthesis of DNA, mRNA, and protein synthesis by interacting with purine bases on gDNA or mitochondrial DNA, inhibiting DNA replication and promoting cell apoptosis (57). CIS is widely used in many types of tumors, such as lung, ovarian, breast, bladder, and testicular cancers, but drug resistance and multi-organ toxicity limit its practical use (58).

A previous study has found that QUE enhanced the growth inhibition of CIS in HepG2 HCC cells by up-regulating p16 expression (59). For oral squamous cell carcinoma (OSCC), QUE inhibited cholesterol metabolism by inhibiting the AGR2/AKT/SREBP2 signaling pathway, enhancing CIS sensitivity to CIS-resistant CAL-27 cells (60). QUE pre-treatment of Tca-8113 and SCC-15 OSCC lines enhanced CIS cytotoxicity by inhibiting AKT and IKKβ phosphorylation, the NF-κB pathway, and expression of the anti-apoptotic protein xIAP (61). In ovarian cancer cells, QUE enhanced the CIS sensitivity of the CIS-resistant cell line SKOV-3/CIS by inhibiting the expression of SOD2, catalase (CAT), GPX1, HO-1, Nrf 2, and PI3K/Akt/mTOR (62). Hasan et al. found that QUE can down-regulate the mTOR/STAT3 signal of the SKOV-3/CIS cell line, make it sensitive to CIS, and promote apoptosis (63). The results of a network pharmacological analysis by Ji et al. suggested that QUE can potentially enhance the sensitivity of CIS to cervical cancer (64). Similarly, QUE reversed CIS resistance in CIS-resistant nasopharyngeal carcinoma cell lines 5-8F and C666-1 by inhibiting YAP protein expression and activating the p-YAP phosphorylation (65). In addition, compared with CIS alone, QUE promoted apoptosis induced by CIS by down-regulating NF-κB levels in osteosarcoma cells MG-63 (66). For the 143B osteosarcoma cell line, QUE enhanced the sensitivity of CIS to 143B cells by down-regulating KRAS expression (67). The same effect was observed in the BC cell line (EMT6) (68).

As an effective chemotherapeutic agent, CIS is used to treat various human cancers. Drug resistance is an important challenge in tumor treatment. Owing to QUE’s multi-target and multi-activity characteristics of QUE, combination therapy with CIS has achieved some success (**Table 3**). Therefore, the co-delivery of QUE and CIS drug delivery systems has attracted increasing attention, which could pave the way for developing novel strategies to overcome CIS resistance.

**Table 3:** Mechanism of QUE enhancing the sensitivity of different tumor cell lines to CIS.

Cancer type	Cell line	Mechanism	Reference
Hepatocellular carcinoma	HepG2	↑P16	(59)
Oral squamous cell carcinoma	CAL-27	↓AGR2/AKT/SREBP2	(60)
Oral squamous cell carcinoma	Tca-8113, SCC-15	↓AKT, IKKβ, NF-κB, xIAP	(61)
Ovarian cancer	SKOV-3	↓PI3K/Akt/mTOR, Nrf2, SOD2, CAT, GPX1, HO-1	(62)
Ovarian cancer	SKOV-3	↓mTOR/STAT3	(63)
Nasopharyngeal carcinoma	5-8F, C666-1	↑p-YAP ↓YAP	(65)
Osteosarcoma	MG-63	↓NF-κB	(66)
Osteosarcoma	143B	↑miR-217 ↓KRAS	(67)

“↑” represents QUE’s promoting effect. “↓” represents QUE’s inhibiting effect.

### 2.4 Paclitaxel

Paclitaxel (PTX) is a natural anti-cancer compound that is a taxane chemotherapeutic drug. It prevents cell division by stabilizing β-tubulin heterodimers in the microtubules, preventing depolymerization, and inhibiting the G2/M phase of the cell cycle, leading to cell death. It is often combined with platinum-based chemotherapy regimens for the treatment of various malignancies (69).

A study found that low-dose QUE combined with PTX had more potent inhibitory effects on the proliferation, cell cycle, and migration of PC-3 prostate cancer cells than PTX alone, whereas this combination promoted apoptosis (70). This effect was also observed in SKOV3, A2780, EFO27, and OVCAR-3 ovarian cancer cells (69, 71). Further mechanistic studies revealed that the synergistic effect was mediated by the down-regulation of ERBB2 and BIRC5 and the up-regulation of Caspase-3 expression (69). Network pharmacology analysis and experimental verification by Yang et al. showed that QUE increased the sensitivity of the PTX-resistant BC cell line MCF-7/PTX to PTX by inhibiting the EGFR/ERK axis (72). Due to hydrophobicity, QUE and PTX’s therapeutic efficacy is limited (73). Wang et al. demonstrated growth inhibition in PTX-resistant A549 lung cancer cells by inhibiting AKT and ERK phosphorylation using a chitosan nanoparticle-coated QUE and PTX co-delivery system (73).

However, the oral administration of PTX remains problematic owing to its low oral bioavailability (<10%), which has been attributed to its poor solubility in digestive fluid, intestinal permeability, first-pass effect, and the abundant presence of drug efflux protein in the gastrointestinal tract (74). Therefore, developing a co-delivery system of QUE and PTX can potentially improve the availability of PTX and inhibit its efflux.

### 2.5 Multidrug resistance

The resistance of cancer cells to various anti-cancer drugs with different structures and mechanisms of action is called Multidrug resistance (MDR). MDR is a major cause of chemotherapy failure. Its mechanism of action mainly includes DNA repair, regulation of drug targets, metabolic modification, and reduction of drug concentration through the up-regulation of ATP-binding cassette (ABC) protein, such as P-gp (ABCB1), MRP1 (ABCC1), and MRP2 (ABCC2) (75). ABC protein, an ATP-driven drug efflux transporter, is distributed on the surface of tumor cells as an efflux pump to reduce the effect of chemotherapy drugs so that tumor cells form a unique defense against chemotherapy drugs (75). Therefore, inhibition of ABC efflux transporter activity or inhibition of its expression is very promising for improving the chemotherapeutic effect of tumors.

The mechanism of the chemosensitizing effect of QUE is complex, and several studies have found that it has a favorable inhibitory effect on MDR (**Table 4**). However, pre-treatment of MDA-MB-231/MDR1 cells with QUE reduced DOX efflux and increased DOX accumulation by down-regulating ABCB1, thereby accelerating cell apoptosis (76). When low concentrations of QUE were used in combination with DOX, QUE increased the accumulation of DOX in MCF-7 and MDA-MB-231 cells by down-regulating the expression of ABCB1, BCRP, and MRP1 and promoted the killing effect of DOX on BC cells, especially BC stem cells (77). Another study combined QUE with DOX, PTX, and vincristine (VCR) on MCF-7 and BC stem cells and confirmed that QUE could down-regulate ABCB1 and inhibit the nuclear translocation of YB-1 to kill BC stem cells when combined with these three drugs (78). QUE reversed the VCR resistance of OSCC KB/VCR cells by inhibiting ABCB1 expression in a concentration-dependent manner (79). In addition, QUE reversed MDR in the leukemia cell line K562/DOX by activating p-JNK and p-p38 and inhibiting p-ERK signaling to inhibit ABCB1 expression (80). Moreover, QUE was found to reverse the MDR of BEL-7402/5-FU cells by inhibiting the expression of ABCB1, ABCC1, and ABCC2 by inhibiting the FZD7/β-catenin pathway (81). In addition, overexpression of CYR61 in AGS cells could up-regulate ABCC1, and QUE treatment suppressed CYR61/ABCC1 expression to suppress MDR (82). Moreover, QUE significantly down-regulated ABCB1 expression in KATOIII/OxR, an oxaliplatin-resistant GC cell line, and increased the apoptosis rate of KATOIII/OxR cells (83). Zhou et al. used metabolomics to detect that QUE increased the sensitivity of DOX-selective ABCB1 overexpression SW620 CRC cells to DOX by inhibiting SLC1A5 expression from antagonizing glutamine metabolism and inhibiting ABCB1 transporter activity (84). MDR1 is also an energy-dependent efflux pump that reduces intracellular drug concentrations and triggers drug resistance (85). QUE combined with gemcitabine and DOX could alleviate chemoresistance of pancreatic cancer AsPC-1 and HCC cells HepG 2 by inhibiting the activity of MDR1 (86).

**Table 4:** The mechanism of QUE inhibiting MDR of different tumor cell lines.

Cancer type	Cell line	Drug	Target	Reference
Breast cancer	MDA-MB-231	DOX	↓ABCB1	(76)
Breast cancer	MCF-7, MDA-MB-231	DOX	↓ABCB1, BCRP, MRP1	(77)
Breast cancer	MCF-7	DOX, PTX, VCR	↓ABCB1	(78)
Oral squamous cell carcinoma	KB	VCR	↓ABCB1	(79)
Leukemia	K562	DOX	↓ABCB1, p-ERK ↑p-JNK, p-p38	(80)
Hepatocellular carcinoma	BEL-7402	5-FU	↓FZD7/β-catenin, ABCB1, ABCC1, ABCC2	(81)
Gastric cancer	AGS	5-FU, DOX	↓ABCC1, p65	(82)
Gastric cancer	KATOIII	Oxaliplatin	↓ABCB1	(83)
Colorectal cancer	SW620	DOX	↓ABCB1, SLC1A5	(84)
Pancreatic cancer, Hepatocellular carcinoma	AsPC-1 HepG 2	Gemcitabine, DOX	↓MDR1	(86)
Breast cancer	MCF-7	DOX	↓ NQO1, MRP1	(87)
Breast cancer	MCF-7	PTX	↓ABCB1	(88)
Breast cancer	MCF-7	DOX	↓ABCB1	(89, 90)
Gastric cancer	SGC7901	DOX	↓Wnt16, ABCB1	(92)
Breast cancer	MCF-7	DOX	↓SRC/PI3K/Akt, ABCG2	(93)

“↑” represents QUE’s promoting effect. “↓” represents QUE’s inhibiting effect.

5-FU, 5-Fluorouracil; DOX, doxorubicin; PTX, paclitaxel; VCR, vincristine.

A phytosome (nano-QUE) prepared with a mixture of QUE and lecithin enhanced DOX-induced apoptosis in MCF-7 BC cells by reducing the expression of NQO1 and MRP1 (87). Liu et al. constructed a QUE co-loaded and chondroitin sulfate-coated mesoporous silica nanoparticles co-delivery system to be absorbed by the CD44 receptor in MCF-7 cells, thereby inhibiting the ABCB1 activity of MCF-7 cells and reducing the efflux of PTX to reverse PTX resistance (88). Another DOX-encapsulated ph-responsive nano-micelle, which is based on chitosan, QUE, and citraconic anhydride, could escape from lysosomes and rapidly release DOX and QUE in the cytoplasm, inhibiting ABCB1 and enhancing the inhibitory effect of DOX on MCF-7/DOX cells (89). Zhang et al. constructed DOX and QUR co-loaded gold nanocages, which have a strong cytotoxic effect on MCF-7/DOX cells by inducing a large amount of ROS production and inhibiting ABCB1 expression (90). ABCB1, which is highly expressed on the surface of intestinal cells, can promote the efflux of DOX, inhibiting DOX from being absorbed by intracellular pathways (91). Mu et al. synthesized QUE-chitosan conjugate, which has high water solubility and opens the tight junctions of Caco-2 colon cells, improving DOX bioavailability and promoting DOX absorption (91). A (HA)-modified silica nanoparticle used to co-deliver QUE and DOX was able to target CD44-overexpressing SGC7901 GC cells, inhibit the expression of Wnt16 and ABCB1, remodel the TME, and reverse MDR (92). A β-cyclodextrin that encapsulates DOX and QUE effectively overcomes the MDR of MCF-7/DOX cells by inhibiting SRC/PI3K/Akt signaling and ABCG2 (93).

**Table 4** illustrates the mechanism by which QUE inhibits MDR to chemotherapeutic agents and demonstrates the potential of QUE in reversing MDR. In conclusion, various nano drug delivery systems enhanced the transcellular transport of DOX and showed inhibitory effects on the ABC protein. Although these drug carrier systems have shown sound inhibitory effects on MDR, they rarely report the toxicity of these drug carrier systems to normal cells or organisms. However, this may need to be further investigated.

### 2.6 Summary

Although various important mechanisms have been found to enhance the sensitivity of QUE to chemotherapeutic drugs, including the regulation of tumor-related signaling pathways and the inhibition of MDR proteins, the diverse causes of drug resistance still need to be further explored. Cancer stem cells (CSC) in the TME are also a key cause of tumor drug resistance (26). Conventional drug therapy mainly targets highly proliferating and mature cancer cells but does not satisfactorily affect quiescent and poorly differentiated CSC cell populations, resulting in tumor cell resuscitation (26). Currently, there is a lack of research on the effects of QUE on CSC, and this may be a new area. Therefore, the effect of QUE combined with chemotherapy drugs on the CSC population is worth exploring in the future, and perhaps a new understanding of the synergistic effect of chemotherapy and QUE. In addition, some studies (94, 95) have found that QUE combined with mycophenolic acid can enhance its anti-cancer effect by increasing the half-life of drugs. However, the effect of QUE on the bioavailability of other chemotherapeutic drugs warrants further investigation in animal models and clinical trials.

## 3 The effect of QUE on the side effects of chemotherapy

Chemotherapy is mainly used for palliative care and adjuvant treatment after surgery. However, chemotherapy is associated with several serious side effects, including early symptoms of toxicity and late symptoms of chronic toxicity (96). According to the WHO classification, its intensity can be mild (grade 1), moderate (grade 2), severe (grade 3), or life-threatening or disabling (grade 4). Immediate toxicity effects can be seen in the skin, hair, bone marrow, blood, gastrointestinal tract, and kidneys. However, all body organs can be affected, including vital organs such as the heart, lungs, and brain. Grade 3 and 4 neurotoxicity can induce somnolence, paresthesia, paralysis, ataxia, spasticity, and coma (96). Additionally, the chronic effects of chemotherapy include drug resistance, carcinogenicity, and reproductive toxicity. However, several preclinical studies have shown that QUE effectively reduces chemotherapy-induced toxicity due to its complex pharmacological activities.

### 3.1 Nephrotoxicity

The key roles of the kidney include the secretion, reabsorption, filtration, bioactivation, and excretion of most substances that enter biological systems, making it a good target for many drugs. Increased and sustained exposure to multiple drugs and drug metabolites can damage the functional units of the kidney, including the vasculature, tubules, and glomeruli. Drug-induced nephrotoxicity is a major cause of acute kidney disease. The toxicity of 5-FU is mainly manifested in liver and kidney toxicity, central nervous system toxicity, and heart toxicity. Ali et al. found that the combination of telmisartan and QUE can significantly reduce the levels of tissue KIM-1, NGAL, cys-C, new inflammatory markers of kidney injury [such as Neutrophil/Lymphocyte (NLP), Monocyte/Lymphocyte (MLR), and Platelets/Lymphocyte ratios (PLR)], and uric acid, which has renoprotective effects against 5-FU-induced nephrotoxicity (97). DOX also causes renal injury, which is mainly characterized by glomerular and tubulointerstitial inflammation and renal fibrosis. In a mice model, urinary creatinine (UCr) was decreased, and 24-hour volume output, creatinine (Cr), urea (UREA), and urine protein were increased after being treated by DOX (98). QUE can up-regulate AKT1, Raf, MEK, and p-ERK signals, restore mitochondrial structure, reduce apoptosis, and inhibit inflammatory factors and Ang II, alleviating DOX-induced kidney injury in mice (98). Under a light microscope, DOX caused renal tubular dilatation, tubular vacuolization, glomerular vacuolization, reduction of Bowman’s space, thickening of Bowman’s capsule, and interstitial infiltration, which were alleviated by QUE treatment (99). Khalil et al. showed that QUE can attenuate DOX-induced elevation of renal nitric oxide (NO), TNF-α, and myeloperoxidase (MPO) activities and ameliorate podocyte injury (100). In addition, QUE can inhibit the accumulation of malondialdehyde (MDA) and the increase in glutathione peroxidase (GPX) levels induced by DOX and CTX in rats and reduce the oxidative damage to the liver and kidney caused by chemotherapy drugs (101). Seker et al. also found that QUE alleviated kidney injury caused by CTX by up-regulating MAPK/ERK phosphorylation and inhibiting NF-κB (18).

The most common side effect of CIS is nephrotoxicity, with acute kidney injury (AKI) as a common manifestation. It has been established that CIS-associated AKI is closely related to the process of ferroptosis, a cell death induced by iron-mediated lipid peroxidation (102). Shi et al. found that QUE alleviated CIS-induced AKI by inhibiting ferroptosis and cupreptosis (102). Tan et al. found that QUE can significantly inhibit LPS-induced secretion of IL-1β, IL-6, and TNF-α from bone marrow-derived macrophages and reduce the Mincle/Syk/NF-κB signaling pathway and Cr, blood urea nitrogen (BUN), IL-1β, IL-6 and TNF-α levels in AKI model rats, alleviating CIS-induced AKI (103). QUE-loaded chitosan nanoparticles were used to reduce IL-18 and KIM-1 levels in renal tissues of CIS-intoxicated rats and improved histological changes such as renal interstitial nephritis, glomerular atrophy, tubular dilatation, cell degeneration, and necrosis caused by CIS (104).

### 3.2 Cardiotoxicity

Cardiotoxicity induced by chemotherapeutic drugs may be a persistent phenomenon. The first is damage to the cardiomyocytes, and this is followed by left ventricular ejection fraction (LVEF), which, if left untreated, can gradually lead to symptomatic heart failure (105). In *in vivo* studies, QUE combined with DOX resulted in less DOX accumulation in AC16 cardiomyocytes and less toxicity in AC16 cells (77). In another experiment, QUE pre-treatment protected primary cardiomyocytes exposed to DOX by inhibiting oxidative stress and improving mitochondrial function by up-regulating 14-3-3γ expression (106). Bmi-1 can directly regulate oxidative stress levels (107). Dong et al. revealed that QUE inhibited apoptosis, mitochondrial dysfunction, ROS production, and DNA double-strand breaks in DOX-induced H9c2 rat cardiomyocytes by inhibiting Bid, p53, and oxidase (p47 and Nox1) and up-regulating the expression of Bcl-2 and Bmi-1. Furthermore, *in vivo* experiments confirmed this effect (107). In addition, the proteomic analysis showed that QUE promoted damage repair of cardiomyocytes by regulating metabolic activation, protein folding, and cytoskeletal remodeling to inhibit the cardiotoxicity of DOX (108). DOX combined with CTX is the most commonly used chemotherapy regimen for TNBC; however, this regimen can cause severe damage to cardiomyocytes. Zhang et al. reported that QUE up-regulated the p-ERK and c-Myc and down-regulated Caspase-3 in H9c2 cells with DOX-CTX treated, which protected cardiomyocytes (50).

The cardiotoxicity of 5-FU can be shown to increase the expression of cardiac proinflammatory cytokines (TNF-α and IL-1β), NF-κB, and Caspase-3 to enhance the apoptosis of cardiac cells, whereas QUE can significantly improve this effect in rats with cardiotoxicity induced by 5-FU (109). Dorostkar et al. used DOX liposomes combined with free QUE to inhibit body weight loss and creatine phosphokinase-MB (CK-MB), lactate dehydrogenase (LDH), and MDA induced by DOX in rats while increasing the activities of GPX, CAT, and superoxide dismutase (SOD) in the left ventricle, which reduces heart tissue damage and cardiomyocyte apoptosis (110). Similarly, in a nude mouse transplanted tumor model of leukemia cells P388, QUE alleviated the myocardial damage caused by DOX by regulating the activities of GPX, SOD, and MDA to clear reactive oxygen species (52). In addition, Zakaria et al. revealed that QUE combined with DOX can reduce the toxicity of DOX in the heart of rats by increasing the expression of PCG-1α, PPARα, and AMPKα2 and down-regulating serum troponin, CK-MB, and creatine phosphokinase (CPK) (111). In rat models of chronic DOX treatment, DOX significantly reduced systolic ventricular septal thickness, LV posterior wall thickness, and LVEF (17). QUE administration in this rat significantly improved LVEF and NT-proBNP levels, alleviating the cardiotoxicity of DOX by alleviating oxidative stress (17). Nrf2 is a major regulator of oxidative stress signaling and is widely expressed in the cardiovascular system, regulating the expression of antioxidant genes and other cell-protective kinases (112). In DOX-induced rat cardiomyopathy models, QUE reduced elevated blood pressure and left ventricular dysfunction, inhibited the increase in CK-MB and LDH, maintained cell membrane integrity, and enhanced myocardial antioxidant capacity by up-regulating Nrf2 expression to restore biochemical and histological abnormalities of the myocardium (112). The cardiotoxicity of CIS can lead to congestive heart failure and sudden cardiac death in patients treated with CIS (113). However, QUE can reduce ROS-mediated mitochondrial damage and inflammation by regulating the Nrf2/HO-1 and p38 MAPK/NF-Bp 65/IL-8 signaling pathways, thereby antagonizing cardiotoxicity induced by CIS (113).

### 3.3 Neurotoxicity

The neurotoxicity of chemotherapeutic drugs manifests as both peripheral and central neurotoxicity. The main symptoms of central toxicity include encephalopathy, posterior reversible encephalopathy syndrome, and cognitive impairment (114). Peripheral neurotoxicity often presents as acrodynia, sensory neuropathy, and sometimes sensory ataxic movement (115). DOX, as a widely used anti-cancer drug, can cause damage to the cerebral cortex and hippocampus. QUE combined with DOX can significantly reduce oxidative stress, neuroinflammation, synaptic plasticity damage, and DNA damage in the cerebral cortex and hippocampus (19). It has been reported that DOX can mediate the production of free radicals in brain tissue, increase lipid peroxidation and protein oxidation, weaken the antioxidant defense system, and aggravate oxidative stress, leading to neuropsychological changes (116). In addition, in a rat model, DOX combined with QUE alleviated corticosterone excess, oxidative stress in brain tissue, and anxiety-depression-like behavior (117).

Peripheral nervous system damage caused by chemotherapy is a side effect of CIS (118). Unay et al. used QUE to intervene in PC12 nerve cells exposed to CIS and found that QUE reduced CIS-induced apoptosis of PC12 cells, changes in mitochondrial membrane potential, and LDH activity (118). Therefore, QUE can potentially reduce CIS-induced peripheral nervous system lesions (118). Reactive oxygen and nitrogen species produced after CIS treatment cause severe damage to the cell membrane of hair cells, leading to irreversible neurological hearing loss (119). In a zebrafish model, QUE mitigated CIS-induced neuronal hair cell apoptosis (120). In a rat model, QUE combined with CIS reduced hair cell loss, vascular stripe, and spiral ganglion degeneration in rats and had a protective effect against CIS (119). Severe painful sensory neuropathy often occurs during PTX treatment, which necessitates the termination of chemotherapy, leading to further deterioration of the quality of life and survival of cancer patients. A study confirmed *in vivo* and *in vitro* that QUE inhibited PTX-induced histamine release from RBL-2 H3 cells and inhibited plasma histamine levels in rats treated with PTX (121). Histamine released by mast cells is a neurotransmitter closely related to pain transduction (122). QUE can dose-dependently increase the threshold of thermal hyperalgesia and mechanical pain in animal models treated with PTX (121). In addition, QUE dose-dependently inhibited increased PKCϵ and TRPV1 expression levels in the spinal cord and dorsal root ganglion of PTX-treated animal models. QUE also inhibited the translocation of PKCϵ from the cytoplasm to the membrane in the spinal cord and dorsal root segments treated with PTX (121).

### 3.4 Hematopoietic and immune system toxicity

Previous studies have reported toxic effects of DOX on hematopoietic and immune systems (123, 124). DOX combined with QUE significantly improved serum lgG, lgM, lgE, and DNA damage in rat models and weakened spleen cell inflammation, apoptosis, and lymphocyte proliferation inhibition (125). In addition, the combination of QUE and DOX significantly enhanced white blood cells and lymphocytes in a rat model (117). One of the important factors that limit the clinical application of CIS is the side effect of myelosuppression, which manifests as a decrease in the number of bone marrow cells. In rats treated with CIS+QUE, QUE increased hematopoietic growth factors (GM-CSF, SCF, and IL-9) and simultaneously inhibited hematopoietic inhibitors (TNF-α and TGF-β1), thereby increasing the differentiation of white blood cells, platelets, neutrophils, erythrocytes, and lymphocytes (126).

### 3.5 Reproductive toxicity

DOX has been reported to have serious effects on the reproductive system, such as impaired sperm production, vacuolar degeneration, abnormal sperm morphology, reduced number of primordial follicles, and follicular morphological variation (127). In an animal experiment, the reproductive toxicity of DOX was shown to reduce the number of follicles, the volume of ovaries and their associated uterus, the number of uterine layers, and the volume of related structures in female rats. At the same time, QUE combined with vitamin E significantly alleviated these adverse effects (128). Similarly, DOX administration reduced spermatogenesis and significantly reduced spermatogenic cells in the rat model. In contrast, QUE administration resulted in significant regeneration of most spermatogenic tubule epithelial cells and a uniform arrangement of spermatogenic cells, indicating that QUE alleviated the testicular toxicity of DOX (129). CIS is also toxic to the testes. In animal models, CIS was observed to cause degeneration of the seminiferous tubule epithelium, cell detachment from the basal membrane, giant cell formation, cell loss, atrophy, vacuolation, and perivascular fibrosis (130). QUE combined with CIS can reduce atrophy, giant cell formation, and vacuolization, thereby improving testicular injury (130).

### 3.6 Cachexia

Cancer often causes cachexia in patients, with approximately 80% of cancer patients with cachexia not surviving, and 20% of cancer-related deaths are attributed to cachexia (131). 5-FU treatment of C26 tumor-bearing mice resulted in weight and muscle mass loss and mitochondrial function damage, while QUE administration alleviated muscle cross-sectional area loss and improved skeletal muscle mitochondrial size and number, which improved chemotherapy-induced skeletal muscle atrophy (132). Chemotherapy drugs can also cause abnormal lipid metabolism and lead to fat loss, which reduces the quality of life of patients with cancer and exacerbates the shortening of survival. CIS has been shown to promote fat degradation and oxidation in mice and impair fat production and lipid deposition (133). Lin et al. found that the intraperitoneal injection of QUE weakened CIS-induced fat loss in tumor-bearing mice (134). Moreover, *in vitro* experiments showed that CIS promoted fat metabolism in 3T3-Ll adipocytes, whereas QUE inhibited this effect (134).

### 3.7 Mucositis and hand-foot syndrome

5-FU inhibits cell division and proliferation; therefore, it non-selectively targets cells with rapidly dividing capacity, such as tumor and mucosal cells (135). Approximately 80% of patients receiving 5-FU develop mucositis, characterized by apoptosis and mucosal degeneration, which limits nutrient intake and drug application (136). In a CRC mouse model of 5-FU-induced colitis, treatment with QUE significantly reduced bloody stool and diarrhea symptoms and restored colon length (28). Further exploration confirmed that QUE reduced the expression of IL-6, TNF-α, and IL-10 in the serum and IL-1β, IL-6, IL-13, and TNF in colon tissue, thereby reducing the inflammatory response (28). A QUE nano-emulsion improved 5-FU-induced colitis by down-regulating NF-κB and HIF-α expression and inhibiting ROS production (137). Histological observations showed that QUE increased villus width, villus diameter, and intestinal muscle thickness, restoring the histopathological changes caused by 5-FU (137). Oral mucositis is a common side effect of 5-FU combination chemotherapy that often leads to oral swelling, erythema, and ulcers, seriously affecting the patient’s quality of life. QUE nanoparticles constructed by Lotfi et al. also alleviated hemorrhage and inflammatory cell infiltration in tongue tissue and inhibited the expression of NF-κB in the oral mucosa (138). Hand-foot syndrome, also known as palmoplantar erythrodysesthesia or acral erythema, is often induced by drugs such as DOX, 5-FU, or capecitabine (139). A collagen matrix incorporated with QUE inhibited the apoptosis of keratinocytes by down-regulating Caspase-3. It increased the antioxidant capacity by down-regulating the levels MDA in the skin tissue, thereby alleviating capecitabine-induced hand-foot syndrome in rats (140).

### 3.8 Summary

Although chemotherapy is associated with serious side effects, it remains the primary treatment option for cancer. As mentioned above, QUE reduces the toxicity of chemotherapeutic drugs (**Table 5**). In terms of the mechanism, QUE is mainly achieved by inhibiting ROS production, anti-oxidation, and normal cell apoptosis due to QUE’s potent antioxidant, anti-inflammatory, and neuroprotective abilities. However, the low water solubility of QUE lowers its oral availability, and more drug delivery systems need to be developed to maximize its bioavailability. In addition, the currently reported effects and mechanisms of QUE inhibition of chemotherapy side effects are limited to animals and cells, and future clinical studies are needed to evaluate its effectiveness and safety further.

**Table 5:** The function and mechanism of QUE in attenuated side effects of chemotherapeutic drugs.

Chemotherapy drug	Mechanism	Function	Attenuated side effect	Reference
5-FU	↓KIM-1, NGAL, Cys-C, NLP, MLR, PLR	↑Antioxidant capacity ↓Serum uric acid	Nephrotoxicity	(97)
DOX	↑AKT1, Raf, MEK, p-ERK, Bcl-2 ↓Bax, Cyt-c	↑Ucr ↓Cr, 24 h urine volume, UREA, Urine albumin	Nephrotoxicity	(98)
DOX	–	↓Renal histological injury	Nephrotoxicity	(99)
DOX	↓TNF-α	↓Cr, UREA, MPO, NO, Renal histological injury	Nephrotoxicity	(100)
DOX, CTX	–	↑GPX ↓MDA	Nephrotoxicity	(101)
CTX	↑MAPK/ERK ↓NF-κB, Bax, Caspase 3, TNF-α, IL-1β	↓BUN, Cr, Uric acid	Nephrotoxicity	(18)
CIS	↑SLC7A11, GPX4, ATP7B, GLS	↑GSH ↓MDA, ROS	Nephrotoxicity	(102)
CIS	↓Mincle/Syk/NF-κB, IL-1β, IL-6, TNF-α	↓Necrotic tubules in the kidney	Nephrotoxicity	(103)
CIS	↑Bcl-2 ↓IL-18, KIM-1, Bax	↓ MDA, Histological injury ↑GSH, SOD	Nephrotoxicity, Reproductive toxicity	(104)
DOX	–	↑A16 cell alive	Cardiotoxicity	(77)
DOX	↑14-3-3γ ↓Caspase-3	↑ SOD, CAT, GSH ↓LDH, MDA, ROS	Cardiotoxicity	(106)
DOX	↑Bcl-2, Bmi-1 ↓Bid, p53, p47, Nox1	↓H9c2 cell apoptosis, Mitochondrial dysfunction, ROS	Cardiotoxicity	(107)
DOX and CTX	↑ERK1/2, c-Myc↓Caspase-3	↓H9c2 cell necrosis, ROS	Cardiotoxicity	(50)
5-FU	↑IL-10 ↓ NF-κB, Caspase-3, TNF-α, IL-1β	↑SOD, CAT, GPX, GSH ↓Troponin, renin, LDH	Cardiotoxicity	(109)
DOX	↑Bcl-2, ↓ NOX1, Rac1, Rac1-GTP	↑GPX, CAT, SOD ↓CK-MB, LDH, MDA	Cardiotoxicity	(110)
DOX	–	↑GSH, SOD	Cardiotoxicity	(52)
DOX	↑AMPKα2, PPARα, PCG-1α	↓Troponin, CK-MB, CPK	Cardiotoxicity	(111)
DOX	–	↑LVEF↓NT-proBNP	Cardiotoxicity	(17)
DOX	↑NRF2,	↑ Left ventricular ↓CK-MB, LDH	Cardiotoxicity	(112)
CIS	↑Nrf 2, HO-1	↓ROS	Cardiotoxicity	(113)
DOX	–	↑GSH, White blood cells, Lymphocytes ↓Corticosterone, MDA, Anxio-depressive-like behavior	Neurotoxicity, Immunotoxic impact	(117)
CIS	↑GAP-43, Synapsin-1	↓PC12 apoptosis, LDH	Neurotoxicity	(118)
CIS	–	↓Hair cell loss, Degeneration of stria vascularis and spiral ganglion	Neurotoxicity	(119)
CIS	–	↓Hair cell apoptosis	Neurotoxicity	(120)
PTX	↓ PKCϵ, TRPV 1	↓Hyperalgesia, mechanical allodynia	Neurotoxicity	(121)
DOX	↓p53, Caspase-3, TNF-α, IL-6	↑CAT, SOD, IgG, IgM, IgE ↓DNA damage	Immunotoxic impact	(125)
CIS	↑GM-CSF, SCF, IL-9 ↓TNF-α, TGF-β1	↓Myelosuppression	Hematopoietic system toxicity	(126)
DOX	–	↑Ovarian follicles, Ovarian volume, Uterus volume, Uterus layers	Reproductive toxicity	(128)
DOX	–	↑TOAC, GPX, Seminiferous tubular germinal epithelium, Spermatogenic cells arranged uniformly	Reproductive toxicity	(129)
CIS	–	↓Germinal epithelium atrophy, Giant cell formation and vacuolization	Reproductive toxicity	(130)
5-FU	↑Cyto-c, BNIP3, MFN1, p/tP65 ↓FIS1, TFAM, PPARG	↑RF, Gonadal fat pad, SDH, mCSA, Muscle mitochondrial size and number	Cachexia	(132)
CIS	↑FAS ↓TNF-α, TBAR, HSL, Fabp4, AMPKγ2, Hadha,	↑Fat accumulation, Total lipid, triglyceride, Adipocyte size	Cachexia	(134)
5-FU	↓IL-6, IL-13, TNF, IL-1β, IL-10	↑Colon length ↓Bloody stool, Diarrhea	Colitis	(28)
5-FU	↓NF-κB, HIF-α	↑Villus width, Villus diameter, Intestinal muscle thickness ↓ROS	Colitis	(137)
5-FU	↓NF-κB	↓Hemorrhage and inflammatory cell infiltration in tongue tissue	Oral mucositis	(138)
Capecitabine	↑Bcl-2 ↓Caspase-3	↑TAC ↓MDA	Hand-foot syndrome	(140)

“↑” represents QUE’s promoting effect. “↓” represents QUE’s inhibiting effect.

5-FU, 5-Fluorouracil; BUN, blood urea nitrogen; CAT, catalase; CIS, cisplatin; CK-MB, creatine phosphokinase-MB; CPK, creatine phosphokinase; Cr, creatinine; CTX, cyclophosphamide; Dox, doxorubicin; GPX, glutathione peroxidase; GSH, glutathione; LDH, lactate dehydrogenase; LVEF, left ventricular ejection fraction; mCSA, muscle cross sectional area; MDA, malondialdehyde; MLR, Monocyte/Lymphocyte; MPO, myeloperoxidase; NLP, Neutrophil/Lymphocyte; NO, nitric oxide; PLR, Platelets/Lymphocyte ratios; PTX, paclitaxel; RF, rectus femoris; SDH, succinate dehydrogenase; SOD, superoxide dismutase; TOAC, total antioxidant capacity; Ucr, urinary creatinine; UREA, urea.

## 4 Immunomodulation effect of QUE in TME

Innate and adaptive immunity in the TME plays a regulatory role in the recognition and killing of tumor cells. The immune escape of cancer cells and immunosuppressive microenvironment are key factors that support tumor progression. Therefore, changing the inhibitory TME and inducing an anti-cancer immune response is an attractive development direction for tumor immunotherapy.

Zhu et al. revealed that QUE up-regulated the expression of IL-2 and IFN-γ and down-regulated IL-10 in a nude mouse transplanted tumor model of TNBC 4T1 cells and increased the ratio of CD4^+^ and CD8^+^ cell differentiation in the transplanted tumor (141). Similarly, Manni found in this model that QUE can significantly increase the infiltration level of natural killer (NK) cells and decrease the infiltration level of Treg cells (142). Further mechanistic studies confirmed that QUE inhibited the content of Treg cells and the secretion of the immunosuppressive factor IL-10 in 4T1 transplanted tumor mice by inhibiting the IL-6/JAK2/STAT3 signaling pathway; however, it enhanced the cytotoxicity of CD8^+^T cells and NK cells, promoting anti-cancer immunity in the TME (143). Administration of QUE, resveratrol, and curcumin in 4T1 tumor-bearing mice increased the recruitment of T cells, reduced the accumulation of neutrophils and macrophages in the TME, and inhibited the development of tumor-infiltrating lymphocytes into immunosuppressive cell subgroups. Specifically, QUE’s drug combination inhibited CD4^+^T cells differentiate to Th2, tumor-associated neutrophils differentiate to N2 type, and tumor-associated macrophages differentiate to M2 type (144). Another study on CRC (145) revealed that QUE targeting CXCL8 inhibited macrophage polarization towards M2 and thus inhibited CRC progression. It helps reverse the dilemma of immunosuppression in TME and makes TME progress toward immune activation (144). When QUE acts on the TME of melanoma, it not only inhibits the proliferation and migration of B16 cells but also increases the number of M1 macrophages and CD8^+^ T cells by inhibiting PDK1/CD47 signals and promoting the secretion of IL-2 and IFN-γ by CD8^+^ T cells, thereby inhibiting the growth of melanoma (146). Myeloid-derived suppressor cells (MDSC) can promote tumor growth in the TME and are potential therapeutic targets (147). QUE inhibited the increase in MDSCs in CRC mouse models (28).

Immune checkpoint inhibitors have opened up new avenues for the treatment of tumors. Combining QUE and anti-PD-1 antibodies can significantly inhibit the expression of PD-L1, IL-4, and IL-6 and raise the expression of CD8a, CD4, CD11b, and IFN-γ in HCC (148). After the intervention of QUE in the H22 HCC cell BALB/c mouse transplanted tumor model, the expression levels of granulocyte colony-stimulating factor and PD-L1 were decreased, and the proportion of CD86^+^ cells was increased. In contrast, the proportion of CD206^+^ cells was decreased, indicating that QUE promoted the polarization of macrophages towards the M1 type (149). At the same time, QUE could inhibit the PD-1/PD-L1 interaction after the intervention of transplanted tumor mice, which increases the expression of CD8, GZMB, and IFN-γ and weakens the inhibitory effect of PD-L1 on T cells (150). In addition, Que inhibited the growth of BC cells by up-regulating the protein levels of IFN-γ-R, p-JAK2, and p-STAT1 and decreased the expression of PD-L1, promoting the differentiation of γδ T cells into Vδ2 T cell subgroups (151).

Jing et al. established the folic acid-modified liposome QUE, which can inhibit the expression of PD-L1 in osteosarcoma by inhibiting JAK2/STAT3 signaling, antagonizing the proliferation and immune escape of osteosarcoma cells (152). QUE-ferrum nanoparticles constructed by Li et al. inhibited the expression of the PD-L1 receptor by inhibiting the phosphorylation of JAK2 and STAT3. They remodeled the extracellular matrix by down-regulating α-SMA, which could capture tumor antigens and deliver them to tumor drainage lymph nodes, increasing the activation of antigen-presenting cells (153). Similarly, Hong et al. constructed a nanoprodrug self-assembled from polyethylene glycol-poly-4-borono-l-phenylalanine conjugating with QUE. The released QUE can reduce the high expression of PD-L1 on the surface of tumor cells, thus promoting the infiltration of cytotoxic T lymphocytes into the TME and enhancing the effect of tumor immunotherapy (154). Microsatellite-stable CRC is resistant to immunotherapies. A nanoformulated co-delivery of QUE and alantolactone can up-regulate dendritic cells’ co-stimulatory signals (MHCII and CD86) to initiate anti-cancer T lymphocyte response and cytokine secretion. In contrast, the number of Treg cells and MDSC was significantly reduced. This QUE drug delivery system changed the inhibitory TME and promoted the systemic memory anti-cancer response (155).

**Figure 2:**
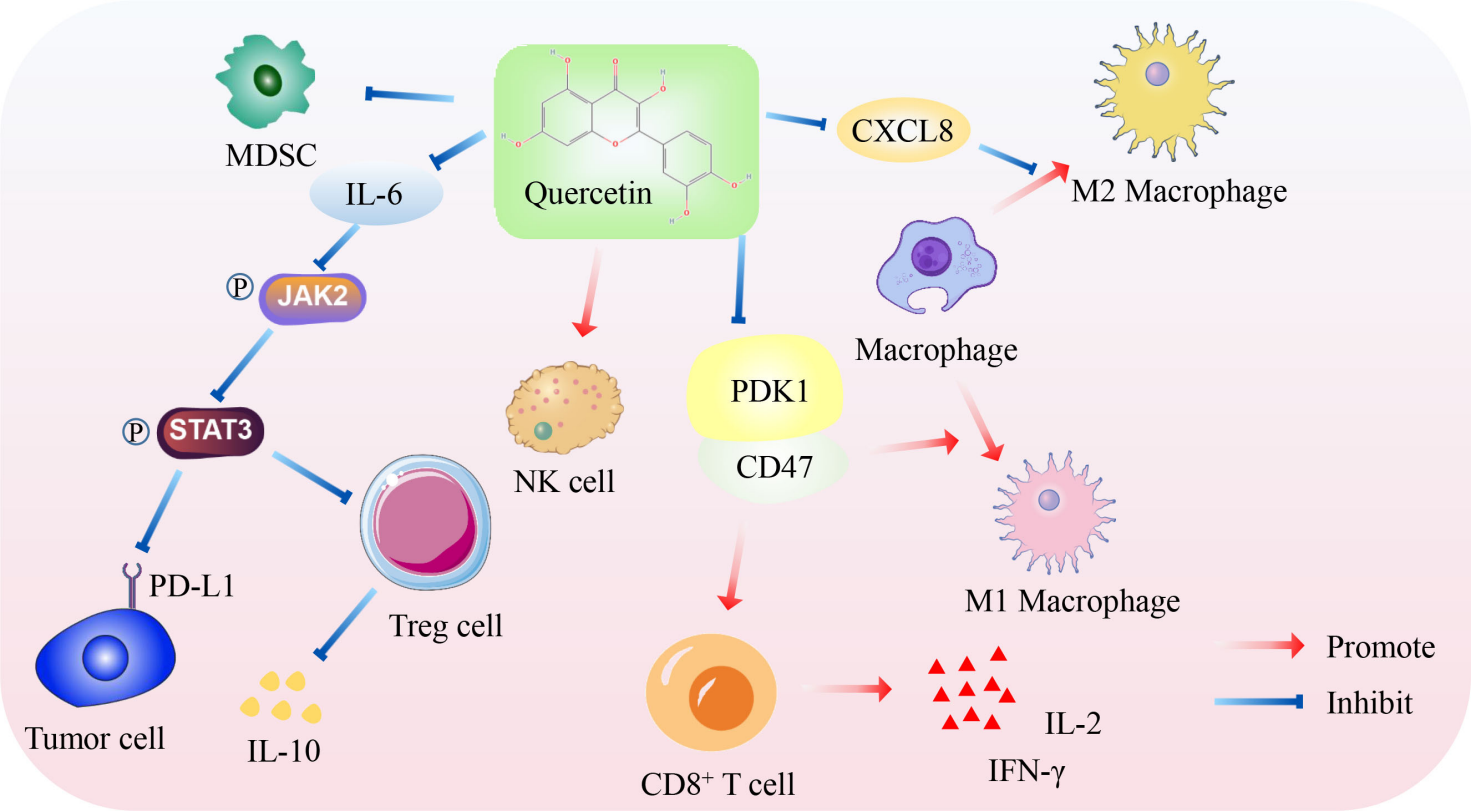
Mechanism of QUE promoting anti-cancer immunity. On the one hand, QUE inhibits PD-L1 expression of tumor cells, Treg cell differentiation, and immunosuppressive factor IL-10 secretion by inhibiting JAK2/STAT3 pathway. On the other hand, QUE inhibits the PDK1/CD47 axis to promote the differentiation of M1 macrophages and CD8^+^T cells and promote the secretion of IL-2 and IFN-γ by CD8^+^T cells. In addition, QUE inhibits immunosuppressive cells, such as M2 macrophages and MDSC, but increases the cytotoxicity of NK cells.

In conclusion, QUE has shown great potential for modulating tumor immune responses. It enhances the anti-cancer immune response by reducing Treg cell differentiation, promoting CD8+T cell differentiation, inducing macrophages to M1-type polarization, and inhibiting the PD-1/PD-L1 immune checkpoint (**Figure 2**). However, these roles and mechanisms need to be further explored and validated. Therefore, QUE may be a potential tumor immunotherapy option in the future.

## 5 Clinical trials

Numerous clinical studies have shown that QUE is beneficial for various diseases such as myocardial infarction (156), upper respiratory tract infection (157), rheumatoid arthritis (158), nonalcoholic fatty liver disease (159), wound healing (160), polycystic ovary syndrome (161), and sarcoidosis (162). Regarding anti-cancer, a clinical study (163) showed that QUE combined with curcumin significantly reduced the size and number of adenomas in patients with familial adenomatous polyposis without showing toxicity to the patients. In addition, a cohort study (164) of two groups of healthy, non-smoking male subjects whose diet was supplemented with QUE or placebo found that plasma TIMP1 mRNA and protein levels were significantly decreased in subjects supplementation with QUE, suggesting that QUE may be a cancer chemopreventive agent. Another prospective randomized controlled study (165) investigated whether QUE could increase green tea polyphenols’ bioavailability in prostate cancer patients. However, QUE did not show any significant effect compared with the placebo. We searched the ClinicalTrials.gov database (https://www.clinicaltrials.gov/) with the keywords “ Quercetin” and “Cancer.” Surprisingly, we found that clinical trials have been completed to investigate the effects of QUE on radiation-induced cystitis, chemotherapy-induced oral mucositis, and cancer-associated Fanconi anemia. In addition, there are several ongoing studies investigating the efficacy and safety of QUE in combination with tislelizumab and dasatinib in patients with head and neck squamous cell carcinoma, the efficacy of QUE and green tea in combination with docetaxel in castration-resistant prostate cancer, and the efficacy of QUE in combination with dasatinib in reversing chemotherapy resistance in TNBC.

We look forward to publishing these clinical trial reports to reveal the potential applications of QUE in cancer. However, there is still a lack of clinical trials focusing on the effect of QUE on chemotherapeutic drugs and the immune status of cancer patients. In the future, more prospective randomized controlled trials with larger sample sizes are needed to explore QUE’s benefits on chemotherapy and cancer patients’ immune status.

## 6 Conclusions and future prospects

Despite significant advances in treatment methods in recent years, cancer remains one of the most serious diseases, causing a large number of deaths each year. Although chemotherapy is considered an effective cancer treatment, its side effects and the development of drug resistance in tumor cells pose significant obstacles to its application. QUE is a widely distributed flavonoid with chemosensitization properties, and it alleviates the toxic side effects combined with various common chemotherapeutic agents. However, the optimal ratio of QUE to chemotherapeutic drugs remains an interesting topic. How to use the proportion of QUE and chemotherapy drugs to achieve an optimal point between anti-cancer effects and reducing toxic side effects may be a problem that needs to be considered in the future. Dormant CSC in tumor tissue escapes the killing of chemotherapeutic drugs and immune cells, which is one of the key factors in chemotherapy resistance and relapse. However, there is still a lack of understanding of the effect of QUE combined with chemotherapeutic drugs on CSC, which is worth exploring. However, existing studies support the idea that QUE promotes an anti-cancer immune response. However, at present, the study of QUE on tumor immunity is in the initial stage, and further multi-omics methods are needed to reveal the influence of QUE on the TME landscape in animal models. The low solubility and bioavailability of QUE and some chemotherapeutic agents limit their application in cancer therapy. Nanoformulation strategies, such as the use of nanoparticles and liposomes, have been implemented, and these strategies have shown excellent results in enhancing chemotherapy sensitivity and mitigating side effects by improving solubility and cellular uptake of QUE and chemotherapy drugs. However, nanocarriers may also be toxic, and attention should be paid to the toxicological properties of nanomaterials. Biodegradable and biocompatible materials should be given a higher priority. Ultimately, more clinical trials are required to evaluate the benefits of QUE in chemotherapy and patient immune status. We believe that with the continuous disclosure of the synergistic effect of QUE with chemotherapy drugs, the mechanism of immune regulation, and the further development of nanomaterials, adjuvant therapy of QUE against tumors will show broad prospects.
